# Electric Bus Pedal Misapplication Detection Based on Phase Space Reconstruction Method

**DOI:** 10.3390/s23187883

**Published:** 2023-09-14

**Authors:** Aihong Lyu, Kunchen Li, Yali Zhang, Kai Mu, Wenbin Luo

**Affiliations:** 1 Vocational and Technical College, Xianyang Normal University, Xianyang 712000, China; lv.aih@163.com; 2 School of Automobile, Chang’an University, Xi’an 710086, China; likunchen@chd.edu.cn; 3 China Academy of Transportation Sciences, Beijing 100029, China; mukai@motcats.ac.cn; 4 Guangzhou Bus Group Co., Ltd., Guangzhou 510098, China; data@gzbsjt.cn

**Keywords:** traffic safety, electric buses, pedal misapplication, phase space reconstruction, deep neural networks

## Abstract

Due to the environmental protection of electric buses, they are gradually replacing traditional fuel buses. Several previous studies have found that accidents related to electric vehicles are linked to Unintended Acceleration (UA), which is mostly caused by the driver pressing the wrong pedal. Therefore, this study proposed a Model for Detecting Pedal Misapplication in Electric Buses (MDPMEB). In this work, natural driving experiments for urban electric buses and pedal misapplication simulation experiments were carried out in a closed field; furthermore, a phase space reconstruction method was introduced, based on chaos theory, to map sequence data to a high-dimensional space in order to produce normal braking and pedal misapplication image datasets. Based on these findings, a modified Swin Transformer network was built. To prevent the model from overfitting when considering small sample data and to improve the generalization ability of the model, it was pre-trained using a publicly available dataset; moreover, the weights of the prior knowledge model were loaded into the model for training. The proposed model was also compared to machine learning and Convolutional Neural Networks (CNN) algorithms. This study showed that this model was able to detect normal braking and pedal misapplication behavior accurately and quickly, and the accuracy rate on the test dataset is 97.58%, which is 9.17% and 4.5% higher than the machine learning algorithm and CNN algorithm, respectively.

## 1. Introduction

Reducing carbon emissions has gradually become a global agenda item in recent decades due to the increasing problems of energy shortages and environmental degradation caused by global climate change and its irreversible effects [1]. Moreover, emissions from motor vehicles are one of the major sources of environmental pollution, making electric vehicles even more encouraging as an alternative solution to conventional fuel vehicles [2,3]. As 70% of the world’s population is expected to live in urban areas by 2050, the development of sustainable urban public transport has become necessary to manage population growth and the significant changes in urbanization [4]. Moreover, countries worldwide are providing strong policy support to decarbonize transport and are considering all-electric buses as an opportunity to improve environmental conditions [5].

According to the National Highway Traffic Safety Administration (NHTSA), 16,000 vehicle accidents in the U.S. each year involve pedal errors while driving, and in Japan, that number is about 6000 [6]. Major accidents involving rear-end collisions, pedestrian injuries, etc. are commonplace, with approximately 12.6% of accidents caused by driver misapplication of the accelerator [7]. Although human hands and feet have similar mechanoreceptor mechanisms, the thresholds of perception for vibrotactile stimuli vary for different body parts. The sensation from the accelerator pedal and brake pedal to the driver’s feet is not as intuitive and sensitive as that from the steering wheel to the palm [8].

Electric buses have no clutch or shift mechanism, and the driver’s handling habits may be different compared with those of traditional fuel vehicles. Referring to a study, when driving a fuel vehicle, before he/she begins the driving cycle, the driver puts the vehicle into forward gear, then gently places his/her right foot on the brake pedal (to prevent the vehicle from “creeping”), and slowly releases the brake to start the vehicle. When driving an electric vehicle, when the driver releases the accelerator pedal, the vehicle stops moving forward. Therefore, during frequent starts and stops, the driver may refrain from putting the right foot on the brake pedal and release the accelerator pedal to be ready to start at any time [9]. In an emergency, the driver will step on the “brakes” more to stop the vehicle; however, the vehicle will be traveling faster (the driver is actually pressing the accelerator pedal). The driver uses the “brake” more, which causes the vehicle to be in a state of rapid acceleration. Often, the driver is unable to judge the situation in the seconds leading up to the crash.

Compared to traditional fuel buses, electric buses exhibit strong starting power and good acceleration performance. In addition, electric buses have higher body mass and can carry more passengers, resulting in more demanding safety requirements. Currently, most electric bus drivers are switching from driving to fuel vehicles [10]. After powering on, the motor of the electric bus can reach its maximum output torque whereas the traditional fuel vehicle engine has a low starting speed and a small output torque is observed; this torque gradually increases as the engine speed. 

Therefore, if an electric bus driver fails to correctly control the pedal during the starting process or presses the wrong pedal due to an emergency, the loss of electric vehicle control will be spontaneous [11]. Research on identifying pedal misuse by electric bus drivers can provide a reference for the design of assisted safe driving systems to avoid “loss of control accidents” caused by drivers mistakenly stepping on the pedals. 

Past research has indicated that improper driver braking behavior and brake system failures are among the primary causes of electric bus crashes; however, it has been obvious that most crashes occur due to the driver’s pedal misapplication [11]. Reference [12] defined the pedal misapplication occurrence as “when the driver is supposed to press a particular pedal (i.e., brake or acceleration) but mistakenly presses the wrong pedal (pedal misapplication) or fails to press any pedal at all (pedal miss)”. In daily driving, drivers typically use the accelerator pedal more often than the brake pedal. Hence, it is even more dangerous for the driver to misuse the accelerator pedal instead of applying the brake [13]. Therefore, the pedal misapplication that this work considers mainly involves the fact that “*the driver should have depressed the brake pedal, but wrongly depressed the accelerator pedal (which may include the driver lifted the accelerator pedal, and then depressed the accelerator pedal again; or directly depressed the accelerator pedal with force, etc.)*”.

Research conducted over the past 20 years has identified several factors that contribute to driver pedal misapplication [13]. These studies have demonstrated the effect of pedal construction design [14], age and gender [15,16,17], shoe contact area with the pedal, single/dual foot operation mode, foot position [18,19], and driver cognitive function [20,21] on pedal misapplication. Currently, studies on pedal misapplication rely mainly on questionnaire surveys [22], analysis of vehicle driving data [23], driver physiological data [15], video-based foot motion trajectory [19], and combinations of vehicle driving data and foot posture data [12,13,19,20]. However, the last three types of data are relatively difficult to acquire during naturalistic driving as they require body posture sensors and medical physiometer devices, which are not usually found in vehicles. Therefore, the objective of this study is to develop a detection method for driver pedal misapplication in electric buses based solely on vehicle driving data, as it only requires a data acquisition device connected to the On-Board Diagnostic (OBD).

Benefiting from significant advancements in computer computing power and the development in the deep learning domain, Convolution Neural Networks (CNNs) are increasingly used in driver behavior recognition tasks. These tasks include braking intention recognition [24], pedal misapplication detection [23,25], driver distraction detection [26,27,28], fatigue detection [29], and so on. However, there is a limited number of studies that have used deep learning to detect pedal misapplication by electric bus drivers, moreover, using the foot camera-based scene data images may encounter problems as they are easily affected by lighting and angle.

Generally, Machine Learning (ML) models are employed to classify problems involving input data presented in the form of time series. However, since entering the 21st century, many more sensors have been deployed in vehicles with the introduction of the information explosion era. Although ML has achieved good results in several engineering domains, the classification performance of traditional ML algorithms decreases with increasing data dimensionality. This behavior results in a “curse of dimensionality” [30]. Compared with traditional algorithms such as random forest and Support Vector Machine (SVM), CNNs can extract deeper and more abstract features. CNNs greatly overcome the limitations of traditional machine learning, such as their limited ability to represent complex functions, weak learning ability, difficulty in parallel computation, and insufficient generalization ability in complex classification problems [31]. CNNs have become a trend to solve various classification and identification problems.

Therefore, the main objective of this work is to develop a CNN-based pedal misapplication detection model, aiming to detect Pedal Misapplication in Electric Buses (PMEB). A natural driving experiment and a closed-field pedal misapplication experiment for urban electric buses were carried out first, and the collected vehicle driving data were processed to manually select the temporal data of the driver’s normal braking and pedal misapplication segments. Based on chaos theory, the phase space reconstruction method was deployed to map the obtained temporal data into high-dimensional color images, producing a driver normal braking dataset and a pedal misapplication dataset.

To sum up, we developed, in this paper, a Model for Detecting Pedal Misapplication in Electric Buses (MDPMEB). Moreover, the main works and contributions of this paper are:

(1) A pedal misapplication detection model for electric buses, based on chaos theory and Swin Transformer structure, was developed. Moreover, the phase space reconstruction method principle, based on chaos theory, was introduced. The network structure of the Swin Transformer and the arithmetic process of the Shifted Windows Multi-Head Self-Attention (SW-MSA) method were presented.

(2) The data collection experiment for electric bus driving was carried out using the CANoe software for data parsing. The time-series data of the bus segments entering a station, passing through intersections and pedestrian crossings, and encountering pedestrians were intercepted.

(3) The experiment of the bus driver’s pedal misapplication in a closed field was conducted, and the dataset of pedal misapplication was established using the phase space reconstruction method.

(4) Pre-training and formal training of the model proposed in this paper were achieved, and finally, the training results were analyzed and compared to the SVM, iForest, ResNet-50, DenseNet, and EfficientNet V2 models.

Finally, this paper is organized as follows: Section 2 presents previous research related to pedal misapplication, whereas Section 3 gives a method to identify pedal misapplication by electric bus drivers. Section 4 presents the results and discussion of this study. The conclusions and deficiencies of this work are delivered in Section 5.

## 2. Related Work

Currently, there are few studies regarding pedal misapplication recognition, which mainly focus on the analysis of pedal misapplication and the design of the system to prevent pedal misapplication. Therefore, in this section, we mainly summarize and sort out the above two aspects.

### 2.1. Pedal Misapplication Analysis and Detection

Kawai and Nakata recruited 44 subjects (both young and old) from Nagoya University to perform a two-handed/bipedal response position selection compatibility task. Participants used their left or right foot (or hand) to press the left or right button according to different computer screen prompts to simulate the process of pressing the accelerator or brake pedal of the vehicle. The experiment found that the driver’s error rate was higher in the bipedal task, the reaction time of the elderly driver was significantly longer when the brake pedal was simulated, and the left dorsolateral prefrontal cortex showed a greater degree of activation [32]. As for Hasegawa et al., they examined the effect of interruptions on the frequency of pedal misapplication by introducing the concept of the “interruption effect”. Moreover, they analyzed the mediating effect of age by recruiting young and older participants to perform a pedal response task accompanied by an interruption task (touching the digits). The findings demonstrated that the longer the interruption time of the pedal task is, the more severe the pedal misapplication will be, particularly among older drivers [16]. 

Moreover, Baharom et al. studied the driver’s response and the pedal’s misuse during emergency braking in Malaysia. The researchers prepared a vehicle equipped with a camera, a force pressure sensor, a light cueing device, an audio cueing device, and Arduino hardware instrumentation to explore the emergency braking response characteristics under normal driving and emergency braking conditions. The results show that the farther the driver’s foot is from the target pedal, the greater the error occurrence will be [18]. Furthermore, based on a video camera, a physiograph, a pedal pressure sensor, and an angular tilt sensor, Wu et al. explored the driver’s motion patterns and the electrophysiological characteristics during emergency brake pedal switching [13]. In addition, three categories of pedal response types (direct hit, corrected trajectory, and pedal error) were analyzed using Functional Principal Component Analysis (FPCA) using the captured video data from the driving simulation experiments to determine the relationship between the foot trajectory and the pedal misapplication. Added to that, Kajiwara & Murata carried out a road driving simulator experiment where the accuracy of the braking response was evaluated using random forest and plain Bayesian predictors. During this experiment, the left foot stance was one of the input parameters, and the findings showed that the driver’s cognitive errors were the main factor contributing to pedal operation errors [20].

Moreover, Bareiss et al. analyzed the crash data from NWVCSS and North Carolina, manually classified the data into normal braking and pedal misuse, and built a classification model using the Bidirectional Encoder Representations from Transformers (BERT) natural language understanding model. Referring to the model of the sequence library, the fine-tuned model had a classification accuracy of 95.7% [23]. 

As for Wu et al., they collected data from naturalistic driving experiments while using video, foot placement, driver characteristics, and driver cognitive function levels, and estimated the probability of foot placement through the use of a repeated polynomial logit model. Then, they classified the types of pedal applications using a random forest model and got an accuracy of about 91.4% [19]. 

Finally, Hernández et al. analyzed the driver’s Electroencephalogram (EEG) before activating the pedal brake and classified the EEG signal using the Support Vector Machine (SVM) and CNNs. The findings showed that the recognition rate of emergency braking intention was 71.1% and 71.8% for SVM and CNN, respectively [24].

### 2.2. Anti-Pedal Misapplication System Design

In order to mitigate driver pedal misapplication, Prasanna et al. developed a safety system called the “Throttle Override Safety System (TOSS system)” designed for electric vehicles. This system addresses the issue of ‘Design Induced Pilot Error’ by separating the drive train from the drive shaft when the driver of an electric vehicle becomes overwhelmed and accelerates the pedal and brakes simultaneously. By interrupting the vehicle’s power and allowing it to slide freely, the TOSS system reduces the number of crashes caused by the wrong pedal being pressed in panic [33]. Similarly, Zheng et al. concluded a study through investigation and concluded that the driver depresses the accelerator pedal slowly and progressively during vehicle start-up; however, during emergency braking, the force applied by the driver on the brake pedal is sharp and rapid [34]. Therefore, Zheng et al. designed a pedal misapplication remediation system for emergency braking that considers the pedal pressure per unit area of the accelerator pedal and the time consumed by depressing the accelerator pedal as the basic points for detection. 

Similarly, Le et al. installed a master cylinder behind the accelerator pedal of the vehicle and collected the oil pressure in the cylinder using a pressure sensor. The researchers also concluded that, when the driver misuses the accelerator pedal as a brake pedal, the operation is fast and forceful. Consequently, when the accelerator pedal master cylinder pressure exceeds a certain threshold, the controller closes the valve of the accelerator pedal master, preventing any further movement of the pedal [6]. Considered together, pedal misapplication has been relatively poorly studied in driver behavior, and many scholars have conducted driving simulator experiments to analyze the factors that lead to pedal misapplication. Moreover, some scholars have also identified the pedal misapplication using machine learning models or deep learning algorithms to predict the driver’s pedal operation patterns as well as the pedal misapplication by capturing the driver’s foot camera. Other scholars have designed remediation systems after pedal misapplication in software and hardware system design.

However, there is a lack of studies that focus on detecting pedal misapplication by electric vehicle drivers, particularly electric bus drivers. Furthermore, medical physiologic instruments are usually not equipped in vehicles, and the images obtained by scene cameras can be more sensitive to light intensity and installation location. Therefore, in this study, based on the natural driving data of pure electric buses, the aim was to produce a pedal misapplication image dataset and construct a model to detect pedal misapplication in pure electric bus drivers by relying on the excellent feature representation capability of the CNNs.

## 3. Our Proposed MDPMEB Algorithm

This section focuses on a brief introduction to the principles of the overall structure of the MDPMEB algorithm, which includes the theory of phase space reconstruction and the main structure of the Swin Transformer.

**Figure 1:**
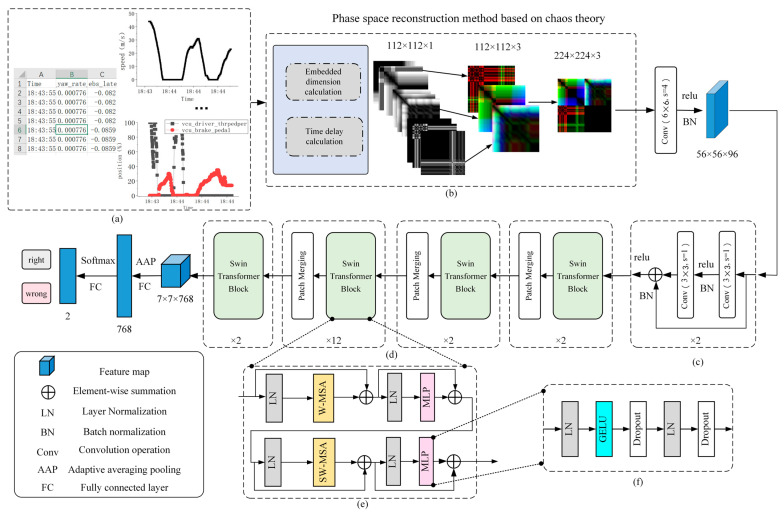
Architecture of MDPMEB. (**a**): driving data (brake segments, each sample has 9 columns of tabular type data.); (**b**): phase space reconstruction module (a mapping method based on time delay method, the input data are changed into gray level map column by column, and then every three columns are superimposed with the output dimension of 112 × 112 × 3 images, and each sample outputs three RGB images, and finally splice them in order.); (**c**): residual network-based downsampling module; (**d**): Swin Transformer skeleton (main structure of Swin Transformer, the numbers below represent the number of this module.); (**e**): Swin Transformer Block module (where W-MSA denotes Windows Multi-Head Self-Attention, SW-MSA denotes Shifted Windows Multi-Head Self-Attention, and MLP denotes Multi-Layer Perceptron); (**f**): MLP structure (containing a series of inactivation and Layer Normalization).

As described in Section 1, the architecture of the model in this paper is illustrated in Figure 1. Figure 1 depicts the approach of mapping time series data to a high-dimensional space, the feature extraction and downsampling modules based on the residual structures, the use of the Swin Transformer structure for self-attentive mechanisms, and provides additional insights into the Swin Transformer Block module and the Multi-Layer Perceptron (MLP) structure. Moreover, the sequence data are mapped to a higher dimensional space, and the RGB images are fed into the residual structure to generate a feature matrix of a specified size. The input consists of a deep multi-layer Swin Transformer network structure, which produces a 7 × 7 × 768 feature map. The results of the driver’s pedal behavior are predicted using adaptive average pooling and fully connected layers.

### 3.1. Phase Space Reconstruction Method Based on Chaos Theory

Natural driving data collected through the vehicle’s OBD and CAN bus has greater convenience and robustness; however, the driving data are recorded in time series format. Although deep learning has excellent feature extraction capability, the tabulated data cannot be directly fed into the CNNs. To weigh the above contradictions, the sequence data were mapped to a higher-dimensional space, and RGB images were generated. Research generally uses the “Takens delay” method for data phase space reconstruction; this method consists of calculating two parameters of optimal embedding dimension and time delay in the time series data to map it to higher dimensional space. After obtaining the above two parameters, the sequence data can be reconstructed using the Recurrence Plot (RP) method, so the problem here is actually how to solve the above two parameters.

In a previous study [35], the authors provided the computational procedure for the “Takens delay” method to obtain the computation. Iterate over all possible values of the embedding dimension and the time delay; first construct the statistic, then solve for the optimal delay time and the optimal embedding dimension. 

Assuming a certain sequence $\left\{x_{i}\right\}\;i=1,2,\ldots ,N$, the reconstructed $R^{m}$ elements can be expressed as follows:

(1) $$X_{k}\left(m,N,\tau \right)=\left(x_{k},x_{k+\tau },\ldots ,x_{k+(m-1)\tau }\right),\;k=1,2,\ldots ,p$$

with

(2) $$p=N-\left(m-1\right)\tau$$

where m represents the embedding dimension (usually 2≤m≤5), τ is the time delay, N indicates the length of the sequence data, and p is the number of vectors in the phase space of the data embedding.

Moreover, assuming that $y_{i}\;\left(i=1,2,\ldots ,O,\;\;O=N-\left(m-1\right)\tau \right)$ is a point in Equation (1), the correlation integral C of the embedded time series is obtained using the below equation.

(3) $$C\left(m,N,r,\tau \right)=\frac{2}{O\left(O-1\right)}\sum_{1\leq i\leq k\leq O}\theta \left(r-{||y_{i}-y_{k}||}_{\infty }\right)$$

(4) $$\theta_{Heaviside}\left(x\right)=\left\{\begin{array}{l} 0,\;x<0\\ 1,\;x\geq 1 \end{array}\right.$$

where r denotes the search radius, it can be taken as any real number in the range of [σ/2,2σ], σ represents the variance of sequence data.

For a certain sequence $\left\{x_{i}\right\}i=1,2,\ldots ,N$, when splitting $\left\{x_{i}\right\}$ into τ non-overlapping time series, the statistic S of the sequence is determined as follows:

(5) $$S\left(m,N,r,\tau \right)=\frac{1}{\tau }\sum_{s=1}^{\tau }[C_{s}(m,\frac{N}{\tau },r,\tau )-C_{s}^{m}(1,\frac{N}{\tau },r,\tau )]$$

Therefore, the extreme deviation ΔS of the statistic S is defined as follows:

(6) $$\Delta S=\max\left[S\left(m,N,r,\tau \right)\right]-\min\left[S\left(m,N,r,\tau \right)\right]$$

Finally, for a particular series, the mean test statistic $\bar{S}\left(\tau \right)$, and the mean statistical extreme deviation $\Delta \bar{S}\left(\tau \right)$ are represented in the below equations:

(7) $$\bar{S}\left(\tau \right)=\frac{1}{n_{m}n_{q}}\sum_{m=2}^{n_{m}+1}\sum_{q=1}^{n_{q}}S\left(m,N,r,\tau \right)$$

(8) $$\Delta \bar{S}\left(\tau \right)=\frac{1}{n_{m}}\sum_{m=2}^{n_{m}+1}\Delta S\left(m,N,r,\tau \right)$$

In addition, combining Equations (7) and (8), $S_{index}$ is determined as follows:

(9) $$S_{index}=\Delta \bar{S}\left(\tau \right)+\left|\bar{S}\left(\tau \right)\right|$$

Referring to the previous studies, the solutions corresponding to the first zero of $\bar{S}\left(\tau \right)$ and the point of the minimal value of $\Delta \bar{S}\left(\tau \right)$ are solved, and the smaller solution obtained from the two equations is considered the optimal delay time $\tau_{d}$. After solving the solution corresponding to the global minimum of $S_{index}$, the optimal embedding dimension $m_{d}$ is calculated based on the $\tau_{d}$. Referring to the above method, we solve for $m_{d}$ and $\tau_{d}$ by writing a piece of Python code. After obtaining the $m_{d}$ and the $\tau_{d}$, the RP method is used to convert the input one-dimensional time series data into a matrix of the corresponding dimension.

### 3.2. Swin Transformer Architecture

The Swin Transformer algorithm was proposed by Microsoft and won the International Conference on Computer Vision (ICCV) 2021 Best Paper [36]. In the paper, the concept of SW-MSA was proposed. In the convolution operation, the feature graph was divided into multiple disjoint regions (windows), and the W-MSA operation was carried out to realize information sharing among different windows, which greatly enhanced the expression ability of the network. Moreover, the self-attention mechanism was proposed in 2017, and the information importance of the input matrix was generated by calculating the weights between each element through a formula [37]. This mechanism is better at capturing the internal correlation of information, has fewer parameters, and is faster to compute. Thus, the algorithm maps the input matrix to a new matrix through Input Embedding and gets the parameter q, k, v through three transformation matrices. Through mutual matching calculation and Softmax processing, the weight of the corresponding input matrix position is obtained. In more detail, the calculation process is described as follows:

(10) $$Attention\left(Q,K,V\right)=softmax\left(\frac{QK^{T}}{\sqrt{d_{k}}}\right)V$$

(11) $$\begin{array}{c} MultiHead\left(Q,K,V\right)=Concat\left(head_{1},head_{2},\ldots ,head_{h}\right)W^{O}\\ where\;head_{i}=Attention\left(QW_{i}^{Q},KW_{i}^{K},VW_{i}^{V}\right) \end{array}$$

where $W_{Q},\;W_{K},\;W_{V}$ represent the parameters trainable transformation matrices and d indicate the length of the input vector.

**Figure 2:**
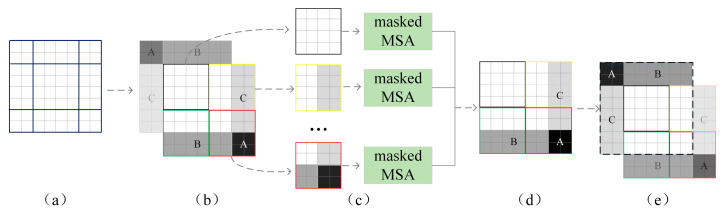
Calculation process of the SW-MSA method. (**a**): Suppose a window with 8 × 8 pixels is divided into nine regions; (**b**): Adjust the position of the shown regions A, B, C respectively, and then re-divide them to 4 × 4 pixel size; (**c**): The MSA calculation is performed on the four regions, respectively, and a new sub-window is obtained, still 4 × 4 in size. (**d**): Splice the sub-windows to obtain a window of size 8 × 8; (**e**): Move the position of regions A, B, C to obtain a new window.

Moreover, the framework of the Swin Transformer is shown in Figure 1d, which consists of several Swin transformer Blocks with different depths and Patch Merging. This latter is used to downsample the input feature matrix, split the feature matrix, and then stitch it according to the position elements. Next, the matrix passes through a LayerNorm block, and finally, it linearly changes through a fully connected layer (which doubles the depth of the feature matrix while reducing the height and width to half of their original size). The structure of the Swin transformer Block is shown in Figure 1e, which mainly consists of W-MSA and SW-MSA. In the MSA method, the input feature matrix is partitioned into several sub-matrices through a Self-Attention operation window, whereas SW-MSA divides the feature map into several regions of different sizes and disjoints, then the different sub-windows are moved and spliced for Self-Attention operation. Finally, the window positions are restored, and the operation process is represented in Figure 2. Note that, in this figure, the input matrix is taken 8 × 8 size as an example after assuming that the window size is 4 × 4; thus, the feature matrix is divided into four windows, and after moving regions A, B, and C, respectively, the masked MSA calculation is performed. Finally, the new 8 × 8 matrix is obtained by performing another split, and the positions of regions A, B, and C are restored.

As shown in Figure 1c, the previous Patch Embedding layer structure is replaced, and the downsampling network is built using the ResNet structure. Moreover, Figure 1f shows the MLP module, which consists of a series of LayerNorm, GELU activation functions, and Dropout deactivation methods.

### 3.3. Training Based on a Priori Knowledge

The number of self-made datasets in this study did not reach the level of tens of thousands; moreover, manual production of datasets is extremely time-consuming. 

To prevent MDPMEB from overfitting during training, which leads to poor model generalization, inspired by the following two studies, we adopt the idea of migration learning (for example, learning to recognize fruits can help identify animals faster). In a study on detecting corn tassels, Liu et al. used the YOLO V5 to detect RGB images captured from a drone [38]. Similarly, in Liu et al.’s study, the number of annotated corn tassel datasets was small (742), and to avoid deep learning limitations, Liu et al. used the VisDrone dataset for pre-training. That is, transfer learning uses the source task as the basis for the target task. In the pre-training process, the target task and the source task are required to be interrelated; that is, the source task belongs to the classification task, and the sample size of the dataset used by the source task must be large enough and the quality good enough [39].

Since the author did not have any self-made dataset that met the conditions, this study adopted the cat and dog dataset from Kaggle as the pre-training dataset, which was agreed to be a good classification dataset. Therefore, the model was pre-trained using a public dataset of dogs and cats, leveraging prior knowledge-based information as the basis for the target task. Alternatively, the pre-training involves using the learned rules as part of the training weights for the target task.

## 4. Experimental Results and Discussion

This section describes the process of conducting the experiment, data preprocessing details, dataset production, and training results of the MDPMEB. It also examines the difference in classification accuracy between the MDPMEB and other classification models. 

Finally, we conducted the experiments using Pytorch-GPU 1.17.0 and Python 3.7 environment implementations on the Ubuntu 18.04 platform. The experimental setup included an i9-12900K CPU and a NVIDIA RTX3090 GPU. The proposed approach was compared to other classical methods and performed both ablation experiments and validation experiments.

### 4.1. Dataset Sources and Collection

This naturalistic driving data collection experiment lasted for about one week in total and was conducted in Guangzhou and Zhengzhou cities in China. Concerning the bus driving data collection experiment realized in Guangzhou City, the study chose route 107, delimited by stations Zhongshan Eighth Road and Flower City Square. The duration of a single run was approximately 80 min, and the operating hours were between 6 a.m. and 9 p.m. Buses on this route run in two shifts (morning and evening), with a total of two drivers involved. Concerning the bus driving data collection experiment realized in Zhengzhou City, the study chose route B1, delimited by stations Power Plant Road and Agricultural Road. The duration of a single run was approximately 60 min, and the operating hours were between 6 a.m. and 9.30 p.m. Buses on this route run in two shifts (morning and evening), with a total of two drivers involved.

Eventually, in this study, the bus travel data were selected based on the experiments in Guangzhou City and Zhengzhou City, with a total of two days and a running time of about 10 h for the first city and a total of four days and a running time of about 40 h for the second city.

**Figure 3:**
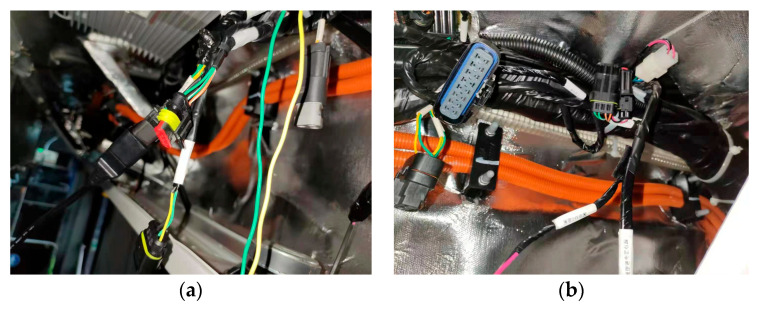
Data acquisition interface of the pure electric bus used in the experiment. (**a**) Electric bus CAN interface; (**b**) OBD interface.

The data acquisition device (Kvaser MemoratorProfessional 5xHS, Produced by Kvaser Company, R&D headquarters is located in Sweden; Source from authorized distributors located in Shanghai, China) was connected to the OBD excuse and the CAN high-voltage/low-voltage interface to record several operating data points of the bus and the vehicle sensor signal data, as shown in Figure 3. The acquisition items include the vehicle ignition system, the gear system, the pedals, the vehicle speed, the braking system, the radar system, the battery pack, relevant sensor signals of the motor group, etc. Moreover, the acquisition frequency of the different modules varies from 10 to 100 Hz.

In the one-week bus-natural driving data collection experiment, stops caused by drivers entering a station, passing through an intersection, passing through a pavement, or other unexpected situations were intercepted into clips. Moreover, they were combined with pedal operation data as well as traveling video data. However, no pedal misapplication by drivers was recorded. Therefore, a simulated driver pedal misapplication experiment in a closed field must be conducted next for further data collection. To obtain more data on pedal misapplication and to consider the safety of the experiment, a real-world experiment was conducted in a closed field to simulate common types of pedal misapplication; moreover, the experimental scheme is shown in Table 1.

The experiment was conducted at a closed test site. A professional driver who has been driving buses for more than 10 years was recruited to drive a bus without passengers and complete the experiment under 4 error types and 3 traveling speeds (as shown in Table 1). In order to reduce the subjective influence of drivers, each experiment was repeated twice. For each experiment, the driver could terminate the state of pressing the wrong pedal after completing the driving behavior of the experimental protocol (Table 1). Then, brake to stop according to your usual driving habits at the end of this experiment and wait for the next experiment. In this study, due to the inherent risks of the experiment, each experiment was only repeated twice at different vehicle speeds, and a total of 24 datasets for different brake pedal misapplications were collected at the end of the experiment. 

After obtaining the natural driving data of the electric bus and the experimental data of the closed site, the braking segments were manually selected under operating conditions such as entering a station, passing through an intersection, and turning. The braking process was defined as the process from braking intention to braking execution to braking hold [10]. However, in our study, to enable earlier identification of pedal misapplication, the training dataset consisted of segments captured 2 s before braking and 1 s after braking. This “braking” is defined as the moment when the driver needs to slow down and starts to press the brake pedal, and when the brake pedal opening is significantly higher (judged by plotting the pedal opening change curve). Of course, in the case of driver error, this “brake” should be the moment when the driver has the braking intention and steps on the accelerator pedal. Therefore, the definition of “brake” may need to be determined on a case-by-case basis. Finally, a piece of raw data were extracted, which means that we obtained a driver braking dataset with a duration of 3 s.

In this study [10], the authors conducted an analysis and found that the most important parameters for the identification of pedal misapplication behavior were the vehicle speed, the motor torque, the accelerator pedal opening, and the brake pedal opening. As part of this study, a total of four index datasets were selected as input. Furthermore, based on previous accidents caused by drivers mistakenly stepping on the pedal, it was obvious that some drivers would steer the wheel randomly due to panic. Therefore, steering wheel data were also considered in this study. During data pre-processing, the bus company may not complete the calibration of the radar system, resulting in low reliability of the data; thus, the data collected by the radar system is also excluded from this research. Therefore, somewhat different from the input selected by Yuan et al., accelerator pedal opening change rate, brake pedal opening change rate, and steering wheel angle data were also selected as model inputs in this study.

Moreover, in this study, 1250 groups of normal braking and 24 groups of pedal misapplication fragments were obtained. The obtained sequence data were mapped to a higher dimensional space to obtain 1250 × 9 gray-scale images. After superimposing each group of brake fragment images, 1253 color images from three channels were obtained. 

However, one may notice that there is an imbalanced number of positive and negative samples in the dataset; however, considering the challenges associated with the safety and cost of repeated closed-site experiments, Generative Adversarial Networks (GANs) may be an effective tool to remedy this problem [40]. GAN includes a G (Generator) and a D (Discriminator). The former is responsible for continuously generating samples similar to the input data, whereas the latter is charged with judging the authenticity of the samples output by the generator. Both play games with each other, and eventually generate new data that is very close to the original. 

Hence, in this study, we built a GAN network, took 24 sets of negative sample images as input, set the training rounds to 500, the learning rate to 0.001, and finally generated 136 images having a size of 224 × 224 × 3. The experimental data were divided into training sets, validation sets, and test sets, as shown in Table 2.

### 4.2. Parameter Settings

**Figure 4:**
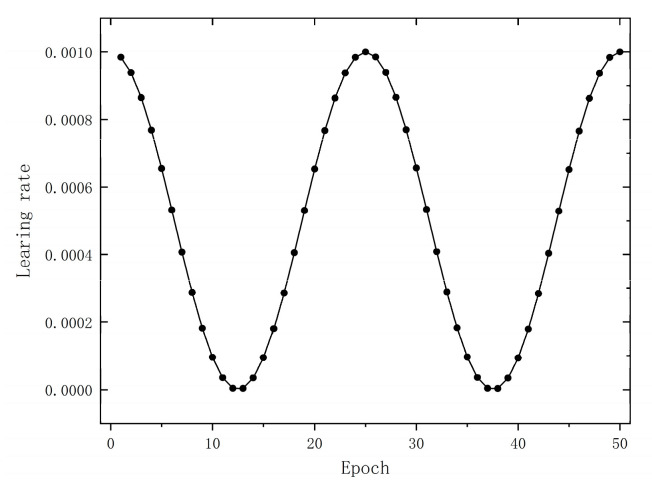
Learning rate change curve.

This section describes the details of the hyperparameter settings and network structure of the MDPMEB model. In more detail, we used a variable learning rate, LambdaLR optimizer, to optimize the loss function; moreover, the learning rate, as a function of the epoch, is shown below, with an initial learning rate of $lr_{0}=0.001$. The curve of learning rate with training batches is shown in Figure 4.

(12) $$lr=\left(\left(1+\cos(4\pi \cdot epochs/50)\right)/2\right)\cdot lr_{o}$$

*L2* regularization adds a penalty term to the loss function to prevent overfitting. Both batches and epochs were used to reduce the computational cost; 64 batches and 50 epochs were deployed. The window size of the W-MSA was set to 7 × 7, and the Embedding depth was set to 96. Therefore, the input matrix size of each layer of the MDPMEB model is shown in Table 3.

### 4.3. Evaluation Parameters

In addition to accuracy, we introduced precision, recall, and fps as the evaluation indexes of the model’s effectiveness, where precision is the proportion of true positive samples predicted by the model with respect to the total predicted positive samples, and recall presents the proportion of true positive samples correctly predicted by the model to the total true positive samples. Finally, the fps indicates the number of frames per second when the model is processed, which reflects the real-time performance of the model. Their expression formulas are shown below:

(13) $$accuracy=\frac{TP+TN}{TP+TN+FP+FN}$$

(14) $$precision=\frac{TP}{TP+FP}$$

(15) $$recall=\frac{TP}{TP+FN}$$

where TP (True Positive) represents the correct positive example, FN (False Negative) represents the incorrect negative example, FP (False Positive) represents the incorrect negative example, and TN (True Negative) represents the correct negative example.

### 4.4. Comparison of Results

We compared this model with other models from two perspectives: first, with traditional machine learning models using sequence data as input, we chose isolation Forest (iForest) and One-Class SVM. Secondly, with the CNN models using images as input, to explore whether the deep learning model can extract more features and improve the performance of the model after converting sequences to images, as well as to verify whether the MDPMEB model with improved Swin Transformer has better performance with other models.

#### 4.4.1. Comparison with the Machine Learning Models

Among the machine learning classification models, iForest requires small data samples and has a linear time complexity, high accuracy, and ability to handle high-dimensional data; moreover, the One Class SVM model is a single classification method that contains only positive samples in the training set. iForest and One-Class SVM models were built, trained, tested, and compared to the MDPMEB. The accuracy, precision, and recall results of the different models were recorded as shown in Table 4.

It should be noted that the input to the machine learning model is canonical sequence data, while the input of the MDPMEB has an image data type. The proposed model outperforms the machine learning model in terms of accuracy, precision, and recall. The accuracy is about 8% higher than iForest and SVM, whereas the recall is about 6% higher than iForest and SVM, and the precision is about 3% higher than both models. The results of this study show that the use of CNNs can indeed improve the recognition accuracy of the model after converting the sequence data to images.

#### 4.4.2. Comparison with Models of Deep Learning

**Figure 5:**
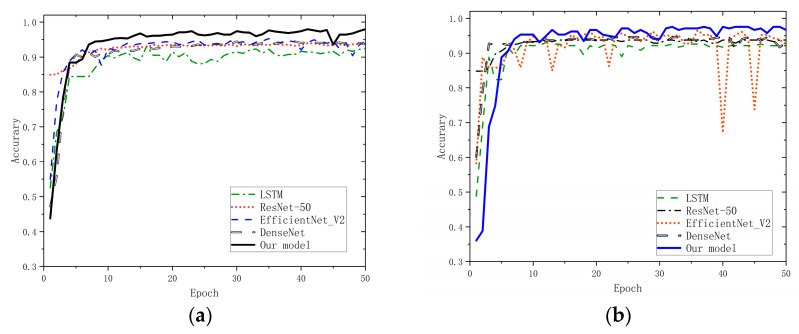
Comparison of accuracy rates during training. (**a**) training dataset and (**b**) validation dataset.

A side-by-side comparison of the mainstream deep learning frameworks is then conducted, where the classical Long Short-Term Memory (LSTM), the Residual Network (ResNet-50), and the EfficientNet V2 were selected to be compared with the proposed model. The accuracy variations of the model in the training process and the validation process are shown in Figure 5.

The results show that: (1) during training, the accuracy of all models except LSTM is around 95% and tends to converge after the 20th iteration; (2) in training and validation, the accuracy of our model improves more slowly compared with LSTM and EfficientNet V2; (3) in training and validation, the stability of the proposed model is the best and its accuracy is the highest (training set: 97.95% and validation set: 95.55%).

**Figure 6:**
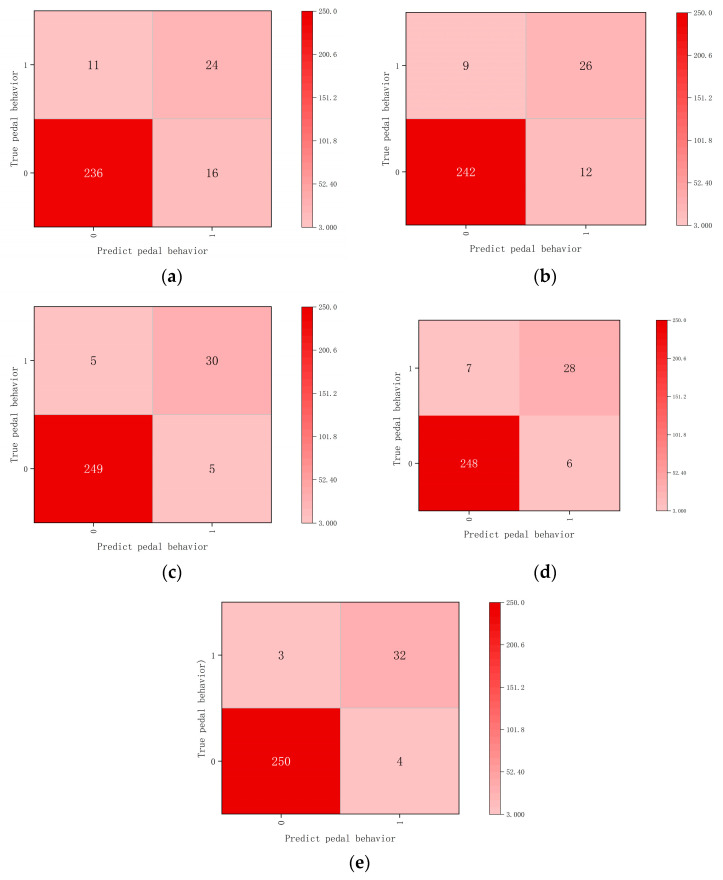
Confusion matrix of the model on the testing dataset, where (**a**): LSTM; (**b**): ResNet-50; (**c**): EfficientNet V2; (**d**): DenseNet; (**e**): the proposed model.

After the training was successfully completed, the models were evaluated using the dataset. Therefore, Table 5 depicts the performance of the models, and Figure 6 shows the confusion matrix of the different models over this dataset.

The results show that: (1) compared to the classical LSTM and ResNet frameworks, the proposed model has excellent performance at the level of accuracy and recall, where the first one is higher than 7.61% and 4.85%, and the second one is higher than 2.88% and 2.01%, respectively; (2) although the difference between the performance of EfficientNet V2, DenseNet, and the proposed model in the test set is not large, our model still performs better than them in the overall performance of check-all rate and accuracy; (3) due to the shallow network depth of LSTM and ResNet and the deeper network layers of the proposed model, we sacrifice a certain operation speed; thus, our model has a lower frame rate of 83 fps when computing the performance of fps; (4) The shallow networks, such as LSTM and ResNet-50, perform poorly in terms of checking the full rate, and they are less effective in identifying the negative samples. Both EfficientNet V2, DenseNet, and the proposed model perform better when recognizing positive samples (normal pedal operation). However, our model also shows a better level of recognition of the negative samples (pedal misapplication).

### 4.5. Ablation Experiment of MDPMEB

The model in this study is based on the ResNet structure and the idea of Swin Transformer, and, based on the original Swin transformer network, part of the layer structure of Swin transformer Block is streamlined, whereas the Patch Embedding layer of the original network is replaced by the ResNet structure. Due to the difficulty of the experimental implementation of real vehicle pedal misapplication, the dataset in this work has unbalanced positive and negative samples. To avoid overfitting the model on less data and to improve its generalization ability to better learn the classification task, a public dataset for pre-training the network layer weights was deployed. SW-MSA is an important idea proposed by Swin transformer architecture, and how much this component contributes to the model is also a question worth discussing. 

**Figure 7:**
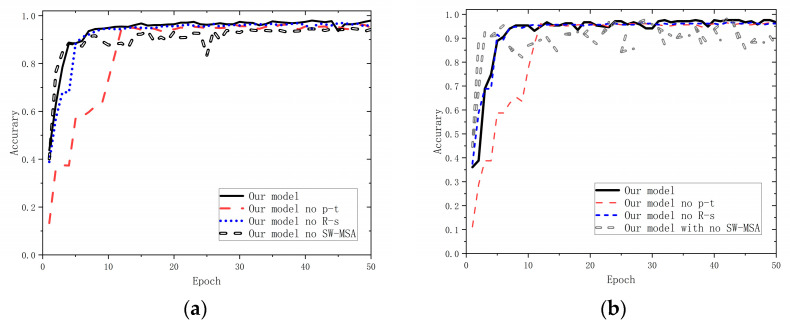
Comparison of accuracy in the training process. (**a**) Training dataset; (**b**) Validation of dataset.

We tested the contribution of the different components by disabling one of the above components of MDPMEB, trained without prior knowledge (our model no p-t), down sampled without ResNet structure (our model no R-s), and without SW-MSA structure (our model no SW-MSA), respectively, and their performance on the training and validation sets is shown in Figure 7. Finally, Table 6 provides the evaluation results of the model when applied to the testing dataset.

Referring to Figure 7, all models can converge after a certain number of iterations, and the accuracy is above 95%, with excellent performance in the verification set. However, after disabling the prior knowledge module, the convergence speed of the model decreases obviously, and the generalization ability decreases in a small range. Moreover, in the training stage, after disabling the prior knowledge weight, no significant gap is encountered in the performance of the optimal accuracy compared to our proposed model, and the performance decreases by about 0.5%. When the residual module is disabled in the early stages of model training, the feature extraction ability of the model slightly decreases; however, this is not very obvious. However, in the verification dataset, the accuracy rate of the model decreases by 1.0% after disabling the residual module. When the SW-MSA method is disabled, the average accuracy in model training decreases by about 2% compared to models with no components disabled. In the validation dataset, as the number of iterations increases, the accuracy of models with SW-MSA disabled fluctuates greatly, and the average accuracy decreases by about 4%.

In the testing phase, the a priori knowledge pretraining, residual modules, and SW-MSA contribute 0.69%, 0.35%, and 2.23%, respectively, without having a significant difference at the level of the accuracy metrics. However, after disabling the prior knowledge preload module, residual structure, and SW-MSA components, the frame rate of the model improved by 2 to 11 frames; however, we still believe that it is meaningful to use these components to improve the work of the model. These findings indicate that, thanks to the powerful data representation capability of Swin Transformer, the use of the prior knowledge-based preloading module and the residual structure down sampling module is beneficial for improving the generalization ability of the model and can prevent it from overfitting on a small dataset.

Unlike previous studies [19,23], this work did not use foot position data and driver physiological data as model inputs but instead used driving data [34] collected by acquisition modules such as OBD or CAN as inputs. Therefore, the optimal accuracy of our model was 1.87% and 6.18% higher than the detection models established by Bareiss et al. and Wu et al., respectively. Meanwhile, our study avoids using cameras or physiologic instruments in the vehicle. Of course, the authors of this article are not denying that the use of video sensors and physiological instruments can help better identify driver behavior; however, in this research, it may not be worth the cost. Compared to a previous study [10], the braking segment changed from 2 s before and after braking to 2 s before and 1 s after braking. However, the accuracy of our model only decreased by 1.92%, which means that the recognition time of pedal misapplication behavior by this model was fully ahead by 1 s.

## 5. Conclusions and Future Work

In this study, we mainly performed PMEB detection, natural driving data collection experiments, and pedal misapplication field experiments for pure electric buses. The driver’s normal braking and pedal misapplication segments of 1253 and 24 groups were manually screened, and a CNN model, based on phase space reconstruction theory, was built to map the time series data to a higher dimensional space in order to produce a pure electric bus driver pedal operation behavior image dataset. This study builds MDPMEB based on the Swin Transformer network and ResNet principles to realize the detection of driver pedal operation misapplication.

Moreover, to prevent the model from overfitting on a small dataset, a pre-training is conducted using the cat-dog public dataset, and the results show that the prior knowledge-based weight preloading module can improve the generalization ability of the model and help to enhance the performance of the PMEB detection task. Moreover, the findings show that combining serialized vehicle driving data with deep convolutional neural networks can quickly and accurately detect pedal misapplication by electric bus drivers. Therefore, based on previous research, this study further explores the analysis of pedal misapplication detection by pure electric bus drivers, which is in line with the current era that promotes bus electrification around the world, and the model can detect normal/abnormal pedal operation of electric bus drivers; thus, a theoretical solution for on-board driver monitoring systems is provided.

There are certain shortcomings in this study. Due to the experimental conditions, the sample size of the dataset in this work is small, and there is a certain problem with an unbalanced dataset. To solve this problem, more drivers could be recruited to carry out pedal misapplication experiments in closed fields. In the closed-field experiment, we were unable to recruit a larger number of drivers within the limited time frame. Therefore, only one professional driver participated and completed the experiment. Although each experiment was conducted twice, it was still challenging to eliminate the influence of subjective factors associated with the driver. Although we use the GAN network to optimize the problem of sample imbalance, it does not fundamentally solve this problem. According to relevant studies, oversampling techniques may be an additional effective method [41].

Another aspect that cannot be ignored is that some sensors may accumulate errors, such as accelerometers, gyroscopes, and radars [42,43]. For instance, Liu et al. discovered that while the Inertial Measurement Unit (IMU) provides independent three-axis acceleration and angular velocity information without relying on external data, it can still lead to an increase in errors within accelerometers due to prolonged integration operations. 

Additionally, Xia et al. designed and developed a data acquisition platform for autonomous vehicles to process multi-source sensor data [43]. They observed that due to the sparsity of radar point cloud data, the obtained sensor data contained certain noises and outliers. To address this, they employed Kalman filters and Chi-square tests for denoising processing. Although we did not utilize point cloud data in this study, outliers may also arise during vehicle operation, such as in speed sensor readings. We did not conduct noise processing during data analysis, which might have had a detrimental impact on the research results.

In future work, we plan to carry out a real vehicle experiment in a closed field again to collect experimental data from different drivers and try to generate small sample data by oversampling or using an improved loss function to further solve the problem of data imbalance. Finally, in this study, we did not consider the influence of drivers’ demographic characteristics (e.g., education level, driving age) on this study. In the future, we can encode driver characteristics and incorporate them into input parameters.

## Figures and Tables

**Table 1:** Closed-field pedal misapplication experiment.

No.	Driver’s Operation	Remarks	Speed (km/h)
1	After releasing the accelerator pedal, depress the accelerator pedal	The driver releases the accelerator pedal and then quickly depresses the accelerator pedal with the strength and speed of an emergency brake for more than 5 s.	10/20/30
2	Pressing the accelerator pedal directly	The driver quickly depresses the accelerator pedal with the force and speed of an emergency brake for more than 5 s.	10/20/30
3	Press the accelerator pedal and brake pedal at the same time with the right foot	Press the accelerator pedal at the same time with the strength and speed of emergency braking for more than 5 s.	10/20/30
4	Press the accelerator pedal with the right foot and the brake pedal with the left foot	The driver’s left and right feet pressed down the brake pedal and accelerator pedal, respectively, vigorously for more than 5 s.	10/20/30

**Table 2:** Number of samples in each class.

No.	Train	Validation	Test
Normal braking	800	199	254
Pedal misapplication	100	25	35

**Table 3:** MDPMEB model architecture.

Step			Input	Output
Conv(6 × 6, s = 4)			224 × 224 × 3	56 × 56 × 96
Module of residual structure (Figure 1c)			56 × 56 × 96	56 × 56 × 96
Swin Transformer Block	stage 1	[window size 7 × 7, head 3] × 2	56 × 56 × 96	28 × 28 × 192
		concat 2 × 2, 192-dim, LN		
	stage 2	[window size 7 × 7, head 6] × 2	28 × 28 × 192	14 × 14 × 384
		concat 2 × 2, 384-dim, LN		
	stage 3	[window size 7 × 7, head 12] × 12	14 × 14 × 384	7 × 7 × 768
		concat 2 × 2, 768dim, LN		
	stage 4	[window size 7 × 7, head 24] × 2	7 × 7 × 768	7 × 7 × 768
FC/AAP		AdaptiveAveragePooling	7 × 7 × 768	1 × 1 × 768
FC/Softmax		LN	768	2

**Table 4:** Comparison of models on the testing dataset.

Model	Accuracy/%	Precision/%	Recall/%	fps
iForest	88.24	94.0	92.52	137.36
One-Class SVM	88.58	95.46	90.95	89.65
Our model	97.58	98.81	98.43	83.78

**Table 5:** Performance of different models on the testing dataset.

Model	Accuracy/%	Precision/%	Recall/%	fps
LSTM	89.97	93.65	95.55	70.42
ResNet-50	92.73	95.28	96.41	103.12
EfficientNet V2	96.54	98.03	98.03	92.81
DenseNet	95.50	97.63	97.25	91.53
Our model	97.58	98.81	98.43	83.78

**Table 6:** Comparison of the evaluation indicators on the testing dataset.

Model	Accuracy/%	Precision/%	Recall/%	fps
Our model no p-t	96.89	98.82	97.67	90.91
Our model no R-s	97.23	98.03	98.81	85.36
Our model no SW-MSA	95.35	97.60	97.21	95.09
Our model	97.58	98.81	98.43	83.78

## Data Availability

The corresponding author can provide data supporting the findings. of this study upon request. Due to ethical or privacy concerns, the data are not publicly available.
